# Formulation of a covalently bonded hydroxyapatite and poly(ether ether ketone) composite

**DOI:** 10.1177/2041731418815570

**Published:** 2018-12-17

**Authors:** Erik AB Hughes, Andrew Parkes, Richard L Williams, Mike J Jenkins, Liam M Grover

**Affiliations:** 1 School of Chemical Engineering, University of Birmingham, Birmingham, UK; 2 NIHR Surgical Reconstruction and Microbiology Research Centre, Queen Elizabeth Hospital, Birmingham, UK; 3 School of Metallurgy and Materials, University of Birmingham, Birmingham, UK

**Keywords:** Poly(ether ether ketone), hydroxyapatite, composite, spinal fusion

## Abstract

Spinal fusion devices can be fabricated from composites based on combining hydroxyapatite and poly(ether ether ketone) phases. These implants serve as load-bearing scaffolds for the formation of new bone tissue between adjacent vertebrae. In this work, we report a novel approach to covalently bond hydroxyapatite and poly(ether ether ketone) to produce a novel composite formulation with enhanced interfacial adhesion between phases. Compared to non-linked composites (HA_PEEK), covalently linked composites (HA_L_PEEK), loaded with 1.25 vol% hydroxyapatite, possessed a greater mean flexural strength (170 ± 5.4 vs 171.7 ± 14.8 MPa (mean ± SD)) and modulus (4.8 ± 0.2 vs 5.0 ± 0.3 GPa (mean ± SD)). Although the mechanical properties were not found to be significantly different (p > 0.05), PEEK_L_HA contained substantially larger hydroxyapatite inclusions (100–1000 µm) compared to HA_PEEK (50–200 µm), due to the inherently agglomerative nature of the covalently bonded hydroxyapatite and poly(ether ether ketone) additive. Larger inclusions would expectedly weaken the HA_L_PEEK composite; however, there is no significant difference between the flexural modulus of poly(ether ether ketone) with respect to HA_L_PEEK (p = 0.13). In addition, the flexural modulus of HA_PEEK is significantly lower compared to poly(ether ether ketone) (p = 0.03). Ultimately, covalent linking reduces hydroxyapatite particulate de-bonding from the polymeric matrix and inhibits micro-crack development, culminating in enhanced transfer of stiffness between hydroxyapatite and poly(ether ether ketone) under loading.

## Introduction

Poly(ether ether ketone) (PEEK) is a high performance semi-crystalline engineering polymer that has been implemented across a range of industry sectors, including oil and gas, electronics, aerospace, automotive and medical.^1–3^ One of the major uses of PEEK in the medical sector is in the fabrication of spinal fusion cages.^2,3^ Spinal cages have been in clinical use since the 1990s and were first shown to be successful in the treatment of horses suffering from nerve root compression.^2,4^ PEEK is an attractive material for this role as it is lightweight, strong and well suited for high load-bearing application.^5–7^ It exhibits a modulus of 3–4 GPa which falls within the range of cancellous and cortical bone (0.05–30 GPa).^2^

Metallic spinal cages, such as those fabricated from titanium alloy (Ti-6Al-4V), are considerably heavier and exhibit a higher modulus (approximately 110 GPa) compared with polymeric counterparts.^8,9^ The modulus mismatch between metal implants and hard tissue can lead to stress shielding, where bone does not experience mechanical stimuli due to the high modulus material bearing a considerable fraction of the applied load. In addition, titanium-based cages are associated with a high occurrence of subsidence for both lumbar and cervical devices, hard tissue weakening and bone porosity development.^10–12^

Improving the ability of PEEK to integrate with bone is recognised as essential for guarantying fusion.^10,13,14^ In a side-by-side study of stand-alone devices, 100% of titanium cages facilitated fusion, whereas PEEK cages were only 76% successful.^10^ PEEK is both hydrophobic and chemically inert, limiting bone attachment and osseointegration compared to Ti-6Al-4V, and increasing the susceptibility of the formation of undesired fibrous tissue about the periphery of implanted PEEK devices.^13,15^

Researchers have extensively developed and characterised PEEK surface porosity and bulk porosity, coatings, surface modifications and composite formulations in order to improve its capacity to support bone formation and integration.^14–25^ It appears that PEEK composite structures are beginning to play a more prominent role as commercially available medical implants as alternatives to metallic materials such as Ti-6Al-4V and stainless steel.^2,17^ While coating technologies enable hydroxyapatite (HA) to be applied to an implant surface, leaving the bulk of the material free from particulate inclusions, such technologies can bring additional processing steps and costs. PEEK composites typically contain bioactive calcium phosphate particulates, including hydroxyapatite (HA, Ca_5_(PO_4_)_3_(OH)_2_) and beta tri-calcium phosphate (β-TCP, Ca_3_(PO_4_)_2_), which have been shown to improve the osseointegration of PEEK with increasing loading level by making surfaces more osteoconductive.^14,16–20,25^ As a reflection of potential bioactivity, apatite mineral has been shown to develop on the surface of HA and PEEK composites following submersion in simulated body fluid (SBF).^26^ Invibio^®^ currently manufactures a medical grade HA and PEEK material, PEEK-OPTIMA^™^ HA Enhanced, which has food and drug administration (FDA) approval for orthopaedic devices and recently acquired the European CE mark of approval.^27^ PEEK-OPTIMA^™^ HA Enhanced devices out-perform PEEK-OPTIMA^™^ Natural devices (HA free) in terms of avoiding fibrous tissue formation, bone on growth and fusion, as demonstrated in a sheep model.^28^

Under loading, however, inclusions can act as stress initiators and risers that diminish the mechanical properties of these materials.^29^ Moreover, dissimilarity between HA and PEEK leads to poor interfacial interactions between the phases, limiting the level of biologically beneficial inclusion. Failure can arise due to HA particulates becoming de-bonded from the polymeric matrix of PEEK.^16,20^ High HA inclusion also increases composite brittleness, as the ductile flow of the matrix is disrupted, decreasing the required energy to initiate fracture.^19,20,30^ HAPEX^™^, a 40% HA in 60% high-density polyethylene blend, is brittle due to high amount of filler; however, it is only bioactive at 40% HA incorporation.^31^ Importantly, only surface exposed HA contributes to enhancement of bioactivity.^26^ Therefore, improving the interfacial adhesion between HA and PEEK may facilitate high inclusion levels of bioactive particulates to augment bioactive performance, without deterioration of physical attributes.

To date, approaches to improve additive adhesion within polymeric matrices have focused on augmenting physical or physiochemical interlocking interactions. Improved mechanical adhesion can be accomplished by in situ polymerisation of PEEK polymer chains in the presence of HA particles, which improves tensile properties up to 30% compared to pure PEEK at 2.6 vol% HA loading.^16^ Silane agents grafted onto filler particles prior to blending with a polymeric matrix can also substantially improve the dispersion and adhesion of additives within PEEK composite formulations.^32,33^ Covalent bonding of HA and PEEK may facilitate even greater interfacial adhesion interactions. Covalently linking PEEK and carbon nanotubes (CNTs) significantly improve the mechanical attributes of resulting composites compared to non-linked counterparts at 1 wt% CNT inclusion.^34,35^ Covalent bonding in these systems was enabled by chemical modification of both CNT and PEEK to afford derivate components that could be directly linked.

The aim of our research is to explore whether covalently bonding HA and PEEK can improve interaction and load transfer between composite phases, potentially enabling greater volumes of HA to be introduced to PEEK-based composites without substantially diminishing mechanical properties. Functionalising HA with the silane (3-mercaptopropyl) triethoxysilane (MPTES) produced HA-SH. Here, silane molecules upon the surface of HA-SH provide a platform for further chemical interactions by presenting reactive thiol groups (–SH) on HA surfaces. Modifying PEEK by a reduction reaction produced PEEK-OH, a chemically accessible derivative previously used in the coupling of CNT and PEEK.^34,35^ These modifications were systematically chosen to enable HA-SH to be covalently linked to PEEK-OH using a heterobifunctional linker, p-maleimidophenyl isocyanate (PMPI). This resulted in a covalently linked HA_L_PEEK additive that could be introduced into a PEEK matrix. Within non-linked (HA_PEEK) and covalently linked (HA_L_PEEK) composites, HA was included at 1.25 vol% in order to focus assessment of the covalent bonding approach at a relatively low loading level. Extensive characterisation of bioceramic and polymer starting materials and respective derivatives is undertaken, as well as physiochemical of analysis of PEEK, and HA_PEEK and HA_L_PEEK composites.

## Materials and methods

Hydroxyapatite (20 µm, ⩾97%, synthetic), (3-mercaptopropyl) triethoxysilane (MPTES) (⩾95%), Propan-2-ol (puriss, p.a., ACS reagent, ⩾99.8% (GC)), HCl (ACS reagent, 37%), potassium hydroxide (reagent grade, 90%), methanol (CHROMASOLV^®^, ⩾99.9%), p-maleimidophenyl isocyanate (PMPI) (purum, ⩾97%) and sodium borohydride (NaBH_4_) (99.99% trace metals basis) were acquired from Sigma-Aldrich Ltd (UK). Ethanol (absolute, analytical reagent grade), dimethyl sulfoxide (DMSO) (analytical reagent grade) and Ellman’s reagent (5,5′-Dithio-bis-(2-nitrobenzoic acid)) were acquired from Fisher Scientific (UK). Acheson Silver DAG was acquired from Agar Scientific (UK). VICTREX^®^ PEEK™ 450PF (25 µm, easy fine flow) was acquired from Victrex plc (UK). Kapton^®^ polyimide film was acquired from DuPont™ (USA). Loctite^®^ Frekote^®^ 44-NC mould release agent was acquired from Henkel (Germany). Distilled water acquired from an arium^®^ advance EDI pure water system by Sartorius (Germany).

### Synthesis and fabrication methods

#### Synthesis of HA-SH derivative

Three vessels were charged 200 mL 90/10 (vol %) propan-2-ol/water solutions. 2 mL MPTES and 250 mg HA were added initially and at 40 minute intervals thereafter under stirring (250 r/min) on a MR stirrer hotplate (Heidolph, Germany). Reaction pH was adjusted to between 3 and 6 at the start of the reaction, and between 9 and 11 after 20 min, and the pattern repeated at 40-min intervals in coordination with the addition of MPTES and HA. pH evolution was manually tracked with a Mettler Toledo SevenCompact™ pH/ion metre equipped with InLab Expert Pro-ISM probe (Mettler Toledo, USA). Stirring was maintained for 4.67 h (seven cycles). HA-SH product was then combined and washed in 5 mL ethanol five times and recovered by centrifugation with a CR4.22 centrifuge (Jouan SA, France) at 4000 r/min for 10 min, before drying at 60°C for 30 min to ascertain full curing of MPTES to HA surfaces.

#### Synthesis of PEEK-OH derivative

PEEK 450PF (5 g) was dispersed in 120 mL DMSO charged with 1.5 g NaBH_4_ under inert argon (Ar) atmosphere. The suspension was heated to 120°C and allowed to react for 24 h, after which the contents were cooled to room temperature. PEEK-OH product was filtered and washed with excess ethanol, distilled water and 0.1 M HCl (diluted from concentrate), then dried at 80°C under vacuum.

#### Synthesis of HA_L_PEEK

HA-SH (5 g) was dispersed in 10 mL DMSO charged with 50 mg of PMPI under constant agitation. After 15 min, 5 g of PEEK-OH was added to the reaction mixture, and the reaction allowed to proceed for a total of 3 h. The resulting product was then washed in methanol, water and methanol again.

#### Composite fabrication and acquisition of test specimens

PEEK, HA_PEEK and HA_L_PEEK powder batches were prepared at a total mass of 50 g. Regarding HA containing batches, the bioceramic content was 1.25 vol% and the polymeric matrix was unmodified PEEK. Prior to processing, batches were kept at 140°C overnight period in order to remove residual moisture. Plaques were fabricated in a Moore Hydraulic Press retrofitted with heating plates (JRD Bipel, UK) to attain temperatures of 400°C. A spacer placed between the plates acted as a frame for the plaque, providing a 27.9 cm^3^ volume. Powders were spread evenly within the press volume and heated to 125°C at minimal plate contact to remove air pockets. Contact pressure was then applied and the temperature increased to 400°C. After 4 h, heating was turned off. Plaques with dimensions of 180 × 150 × 1.2 mm were removed from the press once it had returned to room temperature. Flexural 3-point bending test specimens of 60 × 12 × 1.2 mm were cut out from plaques with a band saw. The span to depth ratio was calculated as outlined in ASTM D790/ISO 178 to ensure specimen failure through compression stress while minimising shear stress.

### Chemical and physical characterisation methods

#### Raman spectroscopy

Raman spectroscopy data were collected using an inVia Raman microscope (Renishaw, UK). The instrument was equipped with a 532 nm laser. Each spectrum was collected over three acquisitions between 100 and 4100 cm^−1^ and the data normalised between sets.

#### Powder X-ray diffraction

Powder X-ray diffraction (XRD) patterns were acquired using a Powder Diffractometer D8 Auto sampler (Bruker, USA) with Cu Kα line (0.154 nm). Pattern data were collected between 2θ values of 5° and 60° with a 0.02° step-size and a step time of 0.5 s/°. Patterns were matched to patterns within The International Centre for Diffraction Data (ICDD) database.

#### Thiol group (–SH) quantification

Quantification of thiol groups was undertaken using an Ellman’s reagent (DTNB, 5,5′-Dithio-bis-(2-nitrobenzoic acid)) assay protocol.^36^ Briefly, a buffer solution was prepared (distilled water, 100 mM Na_3_PO_4_, 1 mM ethylenediaminetetraacetic acid (EDTA), pH 8); 0.05 mL of Ellman’s solution (4 mg DTNB in 1 mL buffer solution) was added to 2.5 mL of buffer solution to produce a reaction solution. Five milligramme of HA-SH was dispersed in a 0.25 mL of buffer solution and added to the reaction solution. The solution was kept agitated for 15 min to develop an assay solution. Upon reacting with free thiol groups, DTNB is converted to 2-nitro-5-thiobenzoic acid (TNB). TNB has a molar absorption coefficient of 14,150 M^−1^ cm^−1^ at 412 nm; 1 mL of this solution was then transferred to a cuvette and the absorbance read at 412 nm with a Cecil CE7500 spectrophotometer (Buck Scientific, USA). The absorbance reading for unmodified HA sample was used as a control and was automatically taken away from the reading acquired from the HA-SH samples. Equations S1–S3 (supplementary information) were followed in order to determine the molar concentration of –SH groups present in the sample.^36^

#### Differential scanning calorimetry

Differential scanning calorimetry (DSC) analysis was undertaken using a DSC 6000 N520-0116 instrument (Perkin Elmer, USA). Approximately 10 mg of sample was held for 2 min at 20°C for temperature stabilisation of the equipment. Samples were then heated to 400°C at a ramp rate of 10°C/min before cooling back down to 20°C at the equivalent ramp rate.

#### Thermal gravimetric analysis

Thermal gravimetric analysis (TGA) was carried out using a STA 449 F3 Jupiter instrument (Netzsch, Germany). Samples were heated to 700°C at a ramp rate of 10°C/min. Further analysis was carried out directly on the data to calculate hydroxylation degree (HD) of the PEEK-OH derivative (equations S4–S9, supplementary information).

#### Scanning electron microscopy

For Figures 4(a)–(d) and 7(a)–(c), specimens were placed upon aluminium stubs using double-sided sticky carbon discs. Specimens were then gold sputter coated using a K550X sputter coater (Quorum Technologies, UK). Scanning electron microscopy (SEM) images were then acquired using an EVO MA 10 scanning electron microscope (Carl Zeiss AG, Germany). For Figure 7(d)–(f), specimen test pieces were placed in liquid nitrogen to allow for cryogenic fracture in order to image a cross-sectional surface. Upon the underside of each specimen, a small amount of silver Acheson Silver DAG was applied in order to reduce charging. Double-sided sticky carbon discs and adhesive were used to secure specimens firmly to aluminium stubs. Specimens were then gold sputter coated using a Polaron SC7640 sputter coater (Quorum Technologies, UK). SEM images were then acquired using a 6060 scanning electron microscope (JOEL, USA).

#### Fourier transform infrared spectroscopy

Fourier transform infrared (FT-IR) spectra were collected using a Nicolet 380 FT-IR spectrometer (Thermo-Scientific, USA), fitted with a Golden Gate ATR attachment (Specac, UK). Measurements were collected between 100 and 4100 cm^−1^ wavenumbers. A background scan was acquired before each scan and subtracted in order to minimise the appearance of H_2_O and CO_2_ molecular modes contaminating each spectrum of interest.

#### Micro-fluorescence spectroscopy (µ-XRF)

Scans were performed in mapping mode on sections of PEEK composites materials with exposed areas of HA using a M4 Tornado instrument (Bruker, USA). Measurements settings of 20 ms/pixel were employed with the instrument operating at 50 kV with anode current of 300 mA. The chamber was maintained at 20 mbar during measurements.

#### Flexural 3-point bend testing

A schematic of the mechanical testing set-up is provided (Figure S1, supplementary information). Tests were performed on a 5566 testing rig (Instron, UK) at a loading rate of 1 mm/min. Flexural strength and flexural modulus were calculated using equations S10–S11 (supplementary information).

### Statistical analysis

One-way analysis of variance (ANOVA) and corresponding Holm–Sidak post hoc tests were performed upon mechanical testing data. Values of p < 0.05 were deemed statistically significant.

## Results and discussion

### Synthesis of HA-SH

**Figure 1.**
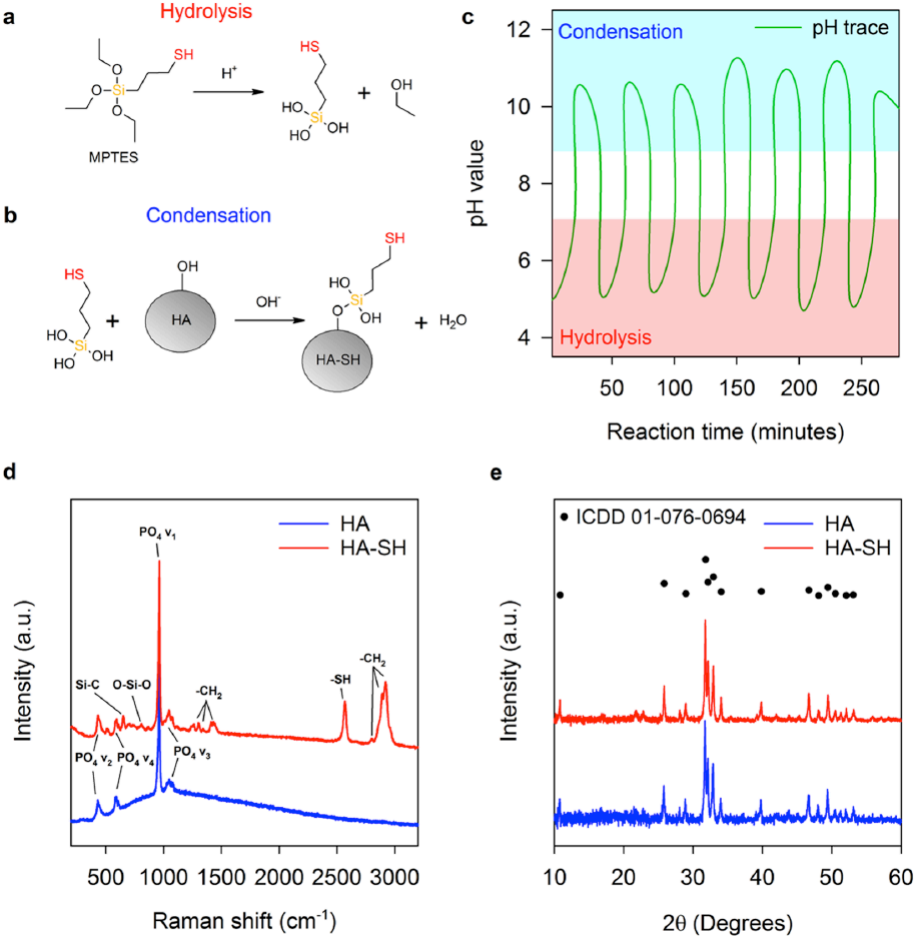
Functionalisation of HA to HA-SH with MPTES. (a) Hydrolysis and (b) condensation reactions of MPTES. (c) Reaction pH evolution. HA and HA-SH (d) Raman spectra and (e) XRD patterns.

MPTES attaches to substrates through hydrolysis and subsequent condensation reactions (Figure 1(a) and (b)). Adjustment of pH over several hours promoted favourable thermodynamic environments for both reactions (Figure 1(c)).^37^ Acidic regions between pH values of 3 and 7 were used to promote hydrolysis of siloxy groups (R-Si-(OCH_3_)_3_) to silanol groups (R-Si-(OH)_3_), and adjustment of the pH to above nine promoted condensation upon HA.

Vibrations of the tetrahedral HA PO_4_ anion, including symmetric stretching (PO_4_ v_1_), symmetric bending (PO_4_ v_2_), asymmetric stretching (PO_4_ v_3_) and asymmetric bending (PO_4_ v_4_), are identified by peaks present at 435, 590, 960 and 1050 cm^−1^, respectively (Figure 1(d)). Carbonate substitution of the HA crystal lattice is suggested by peaks at approximately 1070 cm^−1^ (CO_2_ v_1_).

Peaks relating to MPTES upon HA are present in the HA-SH spectrum (Figure 1(d)), evidencing silane attachment.^36,38–41^ A Si-C stretching peak appears at 652 cm^−1^. A peak at 864 cm^−1^ is present due to CH_2_ rocking. Peaks at 1262, 1301, 1342 and 1431 cm^−1^ are indicative of –CH_2_ twisting modes. Overlapping peaks at 2804, 2891 and 2918 cm^−1^ are due to –CH_2_ vibrations. The –SH stretching peak at 2569 cm^−1^ confirms HA-SH thiol groups.^36^ A Si-O-Si stretching peak located at 809 cm^−1^ indicates silane oligmerisation, suggesting a network of MPTES molecules bound to HA-SH.

Subtle broadening of PO_4_ v_1-4_ peaks in the spectrum HA-SH indicates alterations of P-O bonding environments. Broader peaks indicate structural disorder, while sharper peaks arise from ordered environments.^42^ Differences could be due to the dynamic reaction experienced by HA during modification with MPTES, which may be capable of promoting dissolution and re-precipitation of alternative calcium phosphate phases, as well as the bonding of MPTES to HA-SH. Quantification of thiol groups associated with the surface of HA-SH was approximated at 5.9 × 10^−6^ ± 8.2 × 10^−8^ mol g^−1^.

XRD confirms HA remains the sole bioceramic phase (Figure 1(e)). HA and HA-SH were successfully matched to ICDD pattern number 01-076-0694 (synthetic HA, * quality, with the chemical formula Ca_5_(PO_4_)_3_OH). In addition, crystalline regions of HA are minimally disrupted by the reaction with MPTES as there is no substantial change in crystallinity (77.0% to 76.6%), further evidencing that the widening of PO_4_ peaks in the Raman spectrum of HA-SH is likely due to grafting of MPTES to HA (Figure 1(d)).

### Synthesis of PEEK-OH

**Figure 2.**
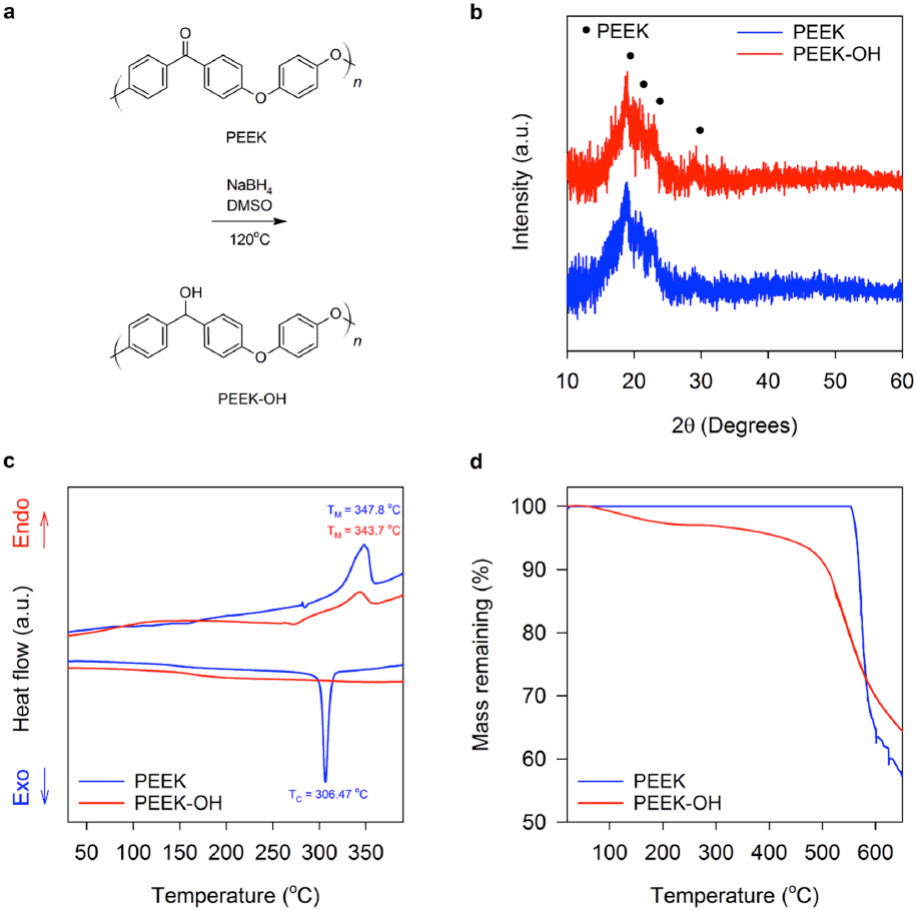
Reduction of PEEK to PEEK-OH with NaBH4. (a) PEEK to PEEK-OH reduction reaction. PEEK and PEEK-OH (b) XRD patterns, (c) DSC traces and (d) TGA traces.

PEEK was converted to PEEK-OH by a reduction reaction (Figure 2(a)).^43,44^ Crystal structures of PEEK and PEEK-OH were assessed by powder XRD (Figure 2(b)). Peaks of both patterns were located at 2θ values of 19°, 21°, 23° and 29°, representative of orthorhombic unit cell PEEK crystal planes of 110, 111, 200 and 211, respectively, suggesting that the lattice parameters are preserved during hydroxylation.^43^ Conversion of PEEK to PEEK-OH reduced crystallinity from 46.1% to 38.4%, as determined by analysis of XRD patterns (Figure 2(b)). Consequently, a reduction in crystallinity can lessen the mechanical properties of the derivative material.^43^ However, PEEK-OH is intended as minor component of HA_L_PEEK (as part of the additive), while the bulk of the composite matrix will be composed of unmodified PEEK polymer. Therefore, we postulate that the reduced mechanical capacity of PEEK-OH will not diminish the overall mechanical properties of HA_L_PEEK compared to HA_PEEK.

The melting temperature (T_M_) of PEEK was found to be 347.8°C, which was approximately 4°C greater than the T_M_ possessed by PEEK-OH of 343.7°C (Figure 2(c)). On cooling, PEEK-OH appears to undergo minimal crystallisation, possessing no clear peak to define crystallisation temperature (T_C_), while PEEK possesses a T_C_ of 306.47°C. This indicates that PEEK-OH becomes fully amorphous due to thermal treatment. Chirality is introduced by the reduction reaction that may contribute to increasing the amorphous nature of PEEK-OH compared to PEEK (Figure S2, supplementary information). The –OH moiety introduced along the polymer chain may also inhibit crystallisation by creating irregularity in forming crystalline regions. Moreover, hydrogen bonding mediated by –OH groups may be extensive enough to suppress polymer chain mobility required for crystallisation.^43^

PEEK undergoes a one-step degradation beginning at approximately 550°C (Figure 2(d)). The steep drop off in mass with temperature exceeding 550°C is indicative of main chain degradation.^45^ PEEK-OH undergoes an initial mass loss between 100°C and 250°C that is attributed to the loss of weakly bound H_2_O molecules. Mass loss between 250°C and 400°C was used to calculate the HD of PEEK-OH. HD of PEEK-OH was 37.6%, consistent with previous work reporting the reduction of PEEK by NaBH_4_ (Figure S3, supplementary information). Main chain degradation begins at approximately 500°C, suggesting that the thermal stability of PEEK-OH is poorer in comparison to PEEK. Promisingly, the polymer derivative remains intact with respect to processing temperatures for PEEK between 380°C and 400°C.

### Synthesis of chemically linked HA_L_PEEK

**Figure 3.**
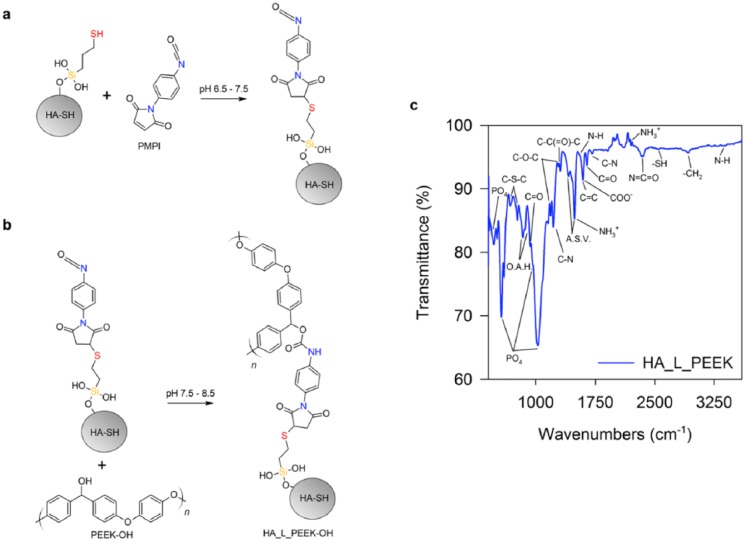
Reduction of PEEK to PEEK-OH with NaBH_4_. (a) Reaction between HA-SH and PMPI chemical linker and (b) between PEEK-OH and PMPI. (c) FT-IR spectrum for HA_L_PEEK.

PMPI possesses maleimide and isocyanate termini. HA-SH thiol groups react with maleimide to produce a thioether bond (C-S-C) (Figure 3(a)). Hydroxyl groups of PEEK-OH react with isocyanate to produce a carbamate bond (R-O-C(=O)-NH-R) (Figure 3(b)).

Newly formed chemical bonds were assessed by FT-IR spectroscopy (Figure 3(c)). PO_4_ modes associated with the structure of HA-SH are located at 470, 608, 962 (as a shoulder) and 1033 cm^−1^. Peaks associated with C-H stretching that originate from MPTES on the HA-SH surface are observed at 2920 cm^−1^.^36,41^ FT-IR confirms formation of C-S-C bonds with PMPI by the symmetric and asymmetric peaks of which are detected at 677 and 771 cm^−1^.^46^ The –SH stretching peak at 2546 cm^−1^ suggests that some thiol groups remain unreacted.^36^

PEEK-OH peaks include out-of-plane aromatic hydrogen (O.A.H.) modes at 840 and 860 cm^−1^, a diphenyl ketone band at 927 cm^−1^, asymmetric C-O-C bending at 1182 and 1278 cm^−1^, C-C(=O)-C bending at 1307 cm^−1^, aromatic skeletal vibrations (ASV) at 1412 and 1493 cm^−1^, and C = O stretching at 1650 cm^−1^. Identification of carbamate bonds was made but was difficult due to peak overlapping.^47–49^ Carbamate COO^–^ and C = O stretching modes expected at 1600 and 1650 cm^−1^ are overlapped by peaks relating to C = C and C = O stretching from PEEK-OH. Peaks for C-N stretching are found at 1220 and 1703 cm^−1^. N-H stretching peaks are located at 1550 and 3370 cm^−1^. Peaks at 1495 and 2194 cm^−1^ are evidence of NH_3_^+^ modes. Unreacted N = C = O groups are identified by the peak at 2341 cm^−1^. Minimal evidence of –OH stretching bands in the region between 3200 and 3550 cm^−1^ also indicates the formation of carbamate bonds, as hydroxyls are utilised during formation of these bonds.

**Figure 4.**
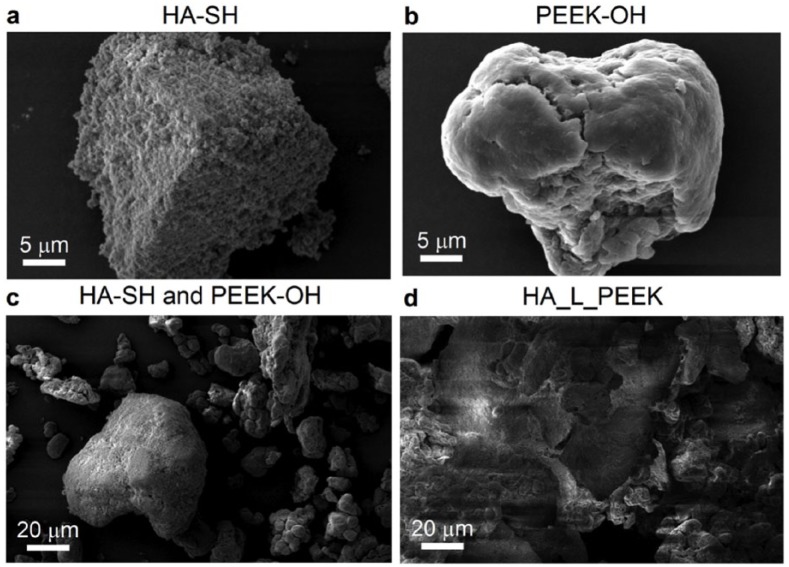
SEM imaging of HA-SH, PEEK-OH and HA_L_PEEK particulates. SEM micrographs of (a) HA-SH, (b) PEEK-OH, (c) a mixture of HA-SH and PEEK-OH particulates and (d) chemically linked HA-SH and PEEK-OH (additive material for HA_L_PEEK composites).

SEM micrographs further evidenced the success of the linking procedure. Figure 4(a) shows that HA-SH particulates are in the approximate size range of between 25 and 50 µm in diameter and furthermore possess textured surfaces and sharp interfaces. Particles of PEEK-OH were between 10 and 30 µm in diameter and possessed smooth surfaces (Figure 4(b)). HA-SH and PEEK-OH powder particulates do not appear to interact strongly when mixed (Figure 4(c)). Mixing alone therefore does not appear to facilitate interactions between the dissimilar phases. After chemical linking with PMPI however, the resulting HA_L_PEEK appears to consist of agglomerates of both powders (Figure 4(d)).

Larger HA-SH particles appear to act as a substrate for bonding interactions with PEEK-OH particles, made possible through the chemical linking procedure. This is also consistent with our experimental method, whereby the maleimide groups of PMPI first react with the –SH groups of HA-SH, followed by the reaction between isocyanate groups of the linker and –OH groups of PEEK-OH when the PEEK derivative is added to the reaction mixture.

### Composite characterisation

**Figure 5.**
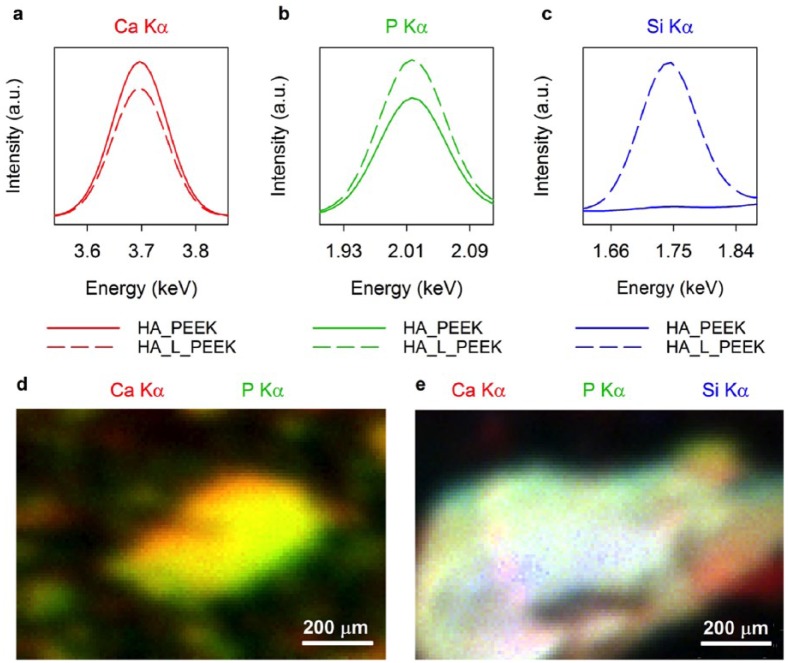
µ-XRF elemental mapping of HA particulates within HA_PEEK and HA_L_PEEK materials following fabrication. (a) µ-XRF spectrum central to the Ca Kα signal. (b) µ-XRF spectrum central to the P Kα signal. (c) µ-XRF spectrum central to the Si Kα signal. (d) Elemental map for a fracture surface of HA_PEEK with Ca and P Kα channels shown. (e) Elemental map for a fracture surface of HA_L_PEEK with Ca, P and Si Kα channels shown.

Chemical linking remained intact following composite fabrication, as confirmed by elemental mapping µ-XRF spectroscopy (Figure 5(a)–(e)). Analysis of the chemical linking chemistry after fabrication was undertaken by comparing elemental maps of HA inclusions within HA_PEEK and HA_L_PEEK composites acquired using a µ-XRF instrument. The main elemental constituents of the HA inclusions were expectedly calcium (Ca) and phosphorous (P) as indicated by Kα signals at 3.7 and 2.0 keV, respectively (Figure 5(a) and (b)). Elemental silicon (Si) was also detected in HA_L_PEEK by a peak at 1.75 keV that was absent in the HA_PEEK spectrum (Figure 5(c)). The silicon signal originates from the presence of the MPTES molecules grafted to HA as part of the chemical linking formulation. Mapping of the Ca Kα and P Kα signals show elemental Ca and P localised to the HA particulates in both composites, in addition to demonstrating the enhanced distribution of HA particles present in HA_PEEK (Figure 5(d) and (e)).

During processing, high temperatures and mobile PEEK chains in the melt are most likely to disrupt and distribute components of the linking chemistry throughout the wider polymeric matrix. Given that the Si content of HA_L_PEEK appears to remain localised to HA particulates (Figure 5(e)), it can be taken as evidence that the chemical linking chemistry remains intact during the processing of composites to provide enhanced interactions between HA and PEEK.

The linking chemistry possessed by HA_L_PEEK was postulated to facilitate the improvement of both the flexural strength and flexural modulus compared to HA_PEEK through the provision of enhanced interfacial interactions between HA and PEEK components greater than mechanical interlocking alone. Mechanical locking interactions can promote brittle failure due to the stiffening effect of HA not being as effectively transferred to the surrounding polymeric matrix, reducing regions of ductile flow about bioceramic irregularities.^19,20,30^ Dense bioceramics, such as HA, possess a relatively high modulus (35–120 GPa) compared to PEEK (3–4 GPa), and therefore, in a perfectly bound HA and PEEK composite, the stiffness should be enhanced.

**Figure 6.**
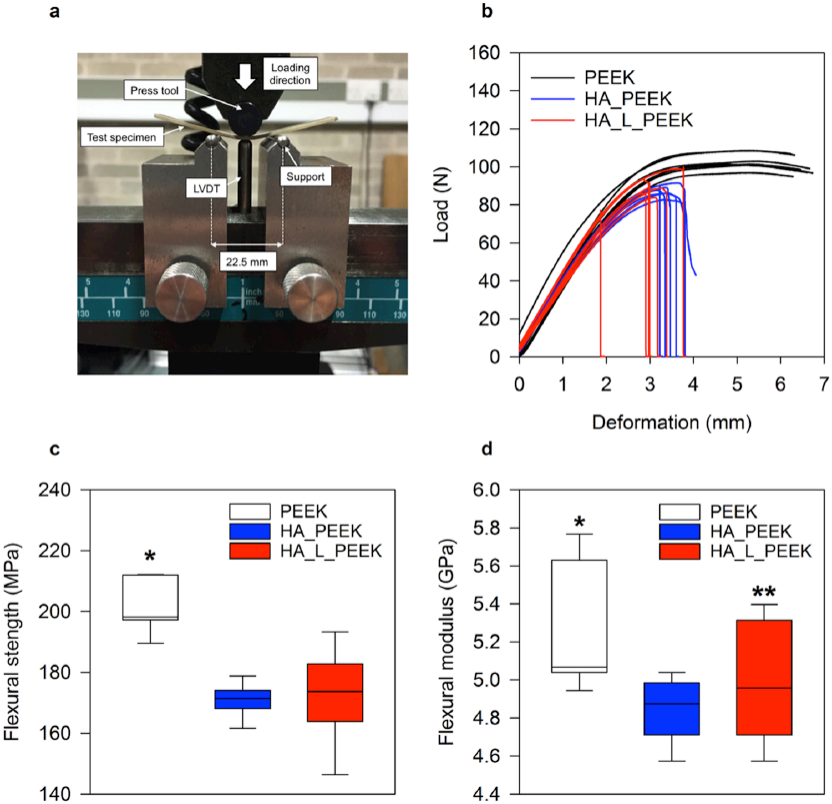
Mechanical properties of PEEK, HA_PEEK and HA_L_PEEK materials. (a) 3-point bend test set-up (LVDT: linear variable displacement transducer). (b) Load–displacement curves for PEEK, HA_PEEK and HA_L_PEEK materials. (c) Flexural strength box-plots for all groups calculated from the load–displacement curves in (b) (n = 7) (*p < 0.05 for PEEK vs HA_PEEK and for PEEK vs HA_L_PEEK). (d) Flexural modulus box-plot for all groups calculated from the load–displacement curves in (b) (n = 7) (*p < 0.05 for PEEK vs HA_PEEK, **p > 0.05 for PEEK vs HA_L_PEEK).

Mechanical properties of the composites were determined from 3-point bend testing (Figure 6(a)) (Figure S1, supplementary information). PEEK specimens underwent ductile failure without fracture and demonstrated plastic deformation at extension beyond the elastic region (Figure 6(b)). PEEK specimens remained whole after testing. Both HA_PEEK (without chemical linking) and HA_L_PEEK (with chemical linking) specimens containing 1.25 vol% HA underwent brittle failure immediately following elastic deformation, resulting in fracture (Figure 6(b)).

PEEK exhibited a flexural strength of 201.2 ± 8.3 MPa (mean ± SD), which was significantly greater than the flexural strength of HA_PEEK and HA_L_PEEK being 170.7 ± 5.4 MPa (mean ± SD) (p < 0.001) and 171.7 ± 14.8 MPa (mean ± SD) (p < 0.001), respectively (Figure 6(c)). Although covalent bonding slightly enhances the mean the flexural strength of HA_L_PEEK compared to HA_PEEK, it is not found to be significantly different (p = 0.85). For all materials, the flexural strength is within the range of 182.9 ± 12.6 reported for unfilled PEEK-OPTIMA^TM^.^50^ The flexural modulus of PEEK was 5.3 ± 0.3 GPa (mean ± SD) (Figure 6(d)). Interestingly, there was no statistical difference between PEEK and HA_L_PEEK materials in terms of flexural modulus, the former possessing a value of 5.0 ± 0.3 GPa (mean ± SD) (p = 0.13). However, the flexural modulus of HA_PEEK, 4.8 ± 0.2 GPa (mean ± SD), was significantly lower in comparison to PEEK (p = 0.03). Although HA_L_PEEK improved upon HA_PEEK in terms of flexural modulus, values were not found to be significantly different (p = 0.30). Flexural modulus values were substantially greater than 2.73 ± 0.26 GPa (mean ± SD) reported for unfilled PEEK, and more comparable to 5.03 + 0.6 reported for VICTREX^®^ PEEK™ 450GL30 (PEEK reinforced with 30% multi-directional chopped glass fibres).^50^ Variation in modulus may arise to due to differences in material processing, namely hot pressing and injection moulding. Importantly, the mechanical properties of HA_L_PEEK appear suitable for load-bearing application, such as that of spinal fusion.

**Figure 7.**
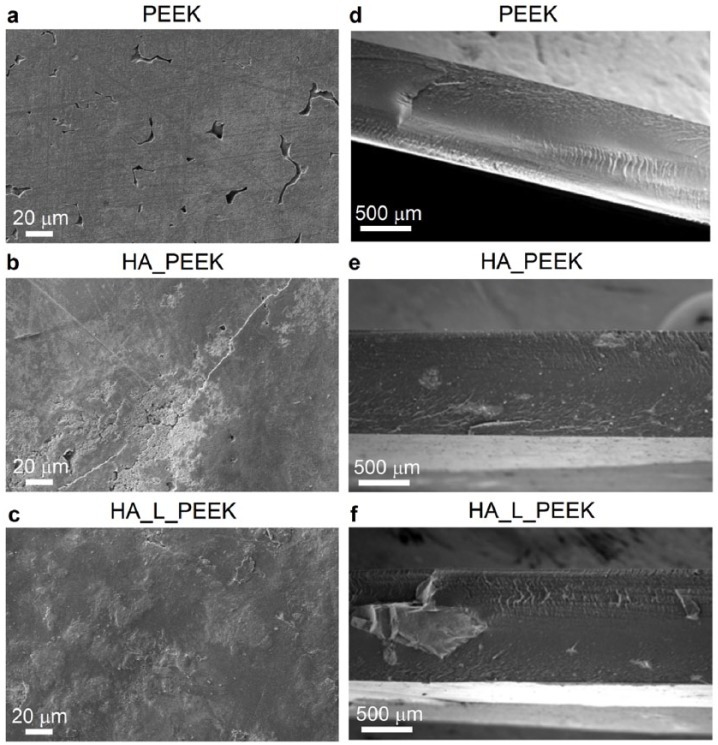
SEM imaging of PEEK, HA_PEEK and HA_L_PEEK materials following fabrication. As fabricated surface of (a) PEEK, (b) HA_PEEK and (c) HA_L_PEEK. Fracture surface of (d) PEEK, (e) HA_PEEK and (f) HA_L_PEEK.

The linking chemistry present in HA_L_PEEK may facilitate the observed improvement in both flexural strength and flexural modulus compared to HA_PEEK through the provision of enhanced interfacial interactions between HA and PEEK components that is greater than mechanical interlocking alone. Positively, covalent bonding interactions between HA and PEEK in HA_L_PEEK appear to lessen the development of HA de-bonding and micro-cracks (Figure 7(a)–(c)). Pre-existing micro-cracks can promote crack initiation, leading to premature failure of materials under load.^51^ HA inclusions within HA_PEEK appear to promote surface visible micro-cracks spanning 10s of microns (Figure 7(b)). HA on the surface of HA_L_PEEK was not associated with de-bonding from PEEK or crack development prior to mechanical testing (Figure 7(c)).

Although significant improvements to the mechanical properties of HA_L_PEEK over HA_PEEK were not attained through covalent bonding between phases, it is important to consider that the mechanical failure of these composites is partly dependent on flaws present within the brittle HA component, as well as the nature of the HA particulates themselves. Augmentation of stiffness is dependent on loading level and the nature of the filler particulates.^29,52^ Lower levels of loading are generally favoured using both micro- and nano-scale particulates, as filler agglomeration that occurs at high loading levels reduces mechanical properties.^25,29,52,53^ This is because as particulate inclusions become smaller, they are less liable to contain flaws, or moreover act as flaws.^51,52,54,55^ Griffith’s law states that the stress concentration at the tip of a defect depends on the defects size.^54,55^ Thus, the combined stress concentration will be greater for and within larger HA particulates.

The HA_L_PEEK additive inherently exists as an agglomeration of chemically bound bioceramic and polymeric particulates, making particle size and dispersion difficult to control in the final composites (Figure 4(d)). Therefore, inclusions were substantially larger within HA_L_PEEK composites, as well as more poorly dispersed throughout the polymer matrix (Figure 7(d)–(f)). The particle size of HA within HA_PEEK is between 50 and 200 µm, and between 100 and 1000 µm within HA_L_PEEK, demonstrating that agglomeration occurred in the processing of both composites, while being exacerbated in the case of HA_L_PEEK. Agglomeration of additive particulates and poor dispersion likely diminishes the mechanical properties of the composites compared to PEEK. However, it is impressive that the introduction of covalent bonding between phases is able to maintain the mechanical properties of HA_L_PEEK composites considering that there is no significant difference in mean flexural strength and modulus between PEEK and HA_L_PEEK materials, while the flexural modulus of HA_PEEK is significantly reduced compared to PEEK.

Ultimately, covalent interactions may improve load transfer between HA and PEEK phases by arresting crack growth and propagation at the interface, benefitting the ability of HA_L_PEEK to resist failure under load by increasing fracture energy.^19,20,30^ Further work will look to reduce inclusion particle size, lessen agglomeration, increase the range of HA loadings and assess *in vitro* cytotoxicity. Refinement of the materials processing and fabrication pathway to overcome issues associated with particle size and agglomeration will enable further investigation of PEEK composites that possess covalently bonded HA at commercially applicable levels and beyond. Utilising nano HA particulates will increase the surface area of interaction between HA and PEEK making it easier to attain homogeneous distribution, potentially allowing higher loadings of bioceramic inclusion to enhance the integration of composites with bone and support the formation of new hard tissue. Validation of these composites as spinal fusion device construction materials may be achieved utilising computer-modelling techniques prior to conducting pre-clinical studies.^56^

## Conclusion

In this article, a novel covalently linked HA and PEEK composite is reported for the first time. Due to the agglomerative nature of the covalently linked HA and PEEK additive, larger HA particulates are found in HA_L_PEEK composites compared to HA_PEEK. This impeded significant enhancement of the mechanical properties of HA_L_PEEK over HA_PEEK composites at 1.25 vol% HA loading. However, covalent bonding appears to improve load transfer between phases by reducing HA particle de-bonding and arresting the development of micro-cracks. Covalently bonding between HA and PEEK may improve the applicability of employing composites derived from these phases.

## Supplemental Material

Supporting_information_EABH_2018 – Supplemental material for Formulation of a covalently bonded hydroxyapatite and poly(ether ether ketone) composite Click here for additional data file. Supplemental material, Supporting_information_EABH_2018 for Formulation of a covalently bonded hydroxyapatite and poly(ether ether ketone) composite by Erik AB Hughes, Andrew Parkes, Richard L Williams, Mike J Jenkins and Liam M Grover in Journal of Tissue Engineering
