# Supplementary material for: Formulation of a covalently bonded hydroxyapatite and poly(ether ether ketone) composite
Source: J Tissue Eng. 2018 Dec 17;9:2041731418815570. doi: 10.1177/2041731418815570 (PMC6299303; doi:10.1177/2041731418815570)
Supplement: Supporting_information_EABH_2018 – Supplemental material for Formulation of a covalently bonded hydroxyapatite and poly(ether ether ketone) composite [file Supporting_information_EABH_2018.pdf]

Supplementary information

Formulation of a covalently bonded hydroxyapatite and poly(ether ether ketone) composite

Erik A. B. Hughes [a,b]*, Andrew Parkes [c], Richard L. Williams [a], Mike J. Jenkins [c], and Liam M. Grover [a]

[a] School of Chemical Engineering, University of Birmingham, Edgbaston, B15 2TT, U.K.

[b] NIHR Surgical Reconstruction and Microbiology Research Centre, Queen Elizabeth Hospital, Birmingham, U.K.

[c] School of Metallurgy and Materials, University of Birmingham, Edgbaston, B15 2TT, U.K.

*Corresponding author: e.a.b.hughes@bham.ac.uk

Figures

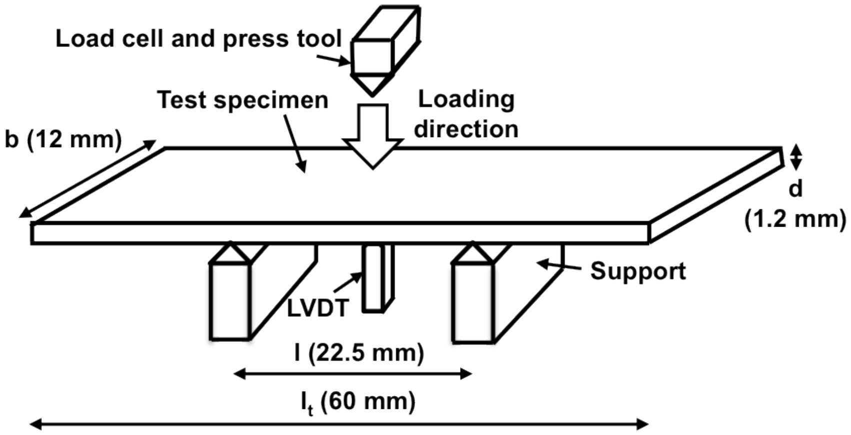

Figure S1 Schematic of mechanical 3-point bend testing set-up, where b = specimen width, d = specimen thickness, l = separation length between supports, l_t = total specimen length, and LVDT = linear variable displacement

transducer. Specimen dimensions and support separation distance comply with ASTM D790/ISO 178 standard.

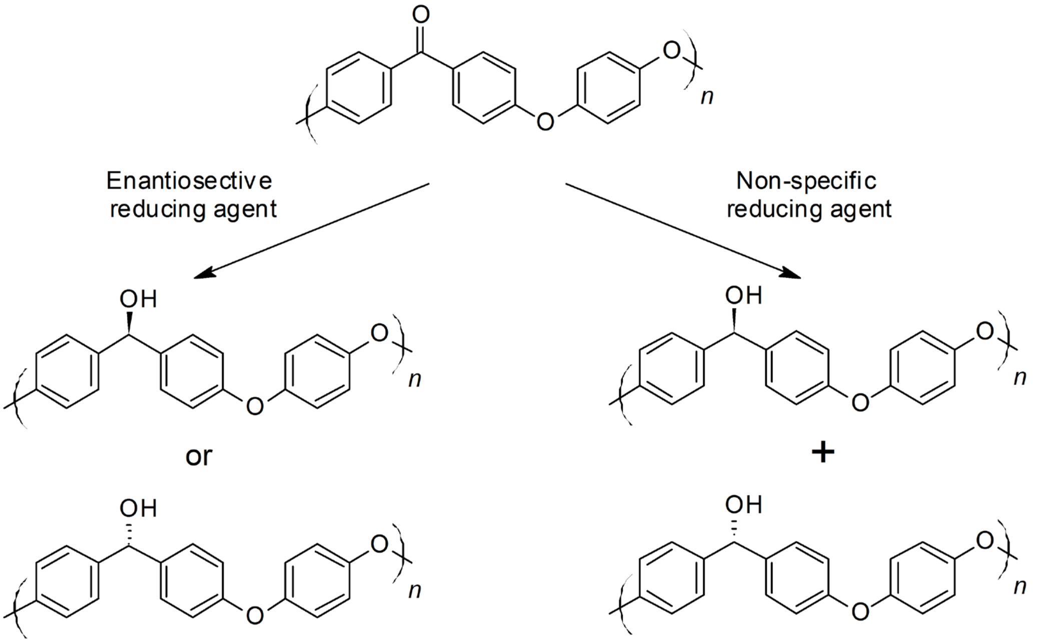

Figure S2 PEEK reduction to PEEK-OH by entioselective and non-specific reducing agents

Chirality is introduced when PEEK is chemically modified by reducing agents facilitating the conversion of =O groups to –OH groups. An enantioselective reducing agent may produce only one enantiomer of PEEK-OH, whereby the –OH groups are configured in one direction. Because $NaBH_4$ is non-selective, a mixture of PEEK-OH enantiomers is expected. The mixture of resulting –OH group configurations may disrupt forming crystallites of the material leading to an increase in the amorphous nature of PEEK-OH compared to PEEK.

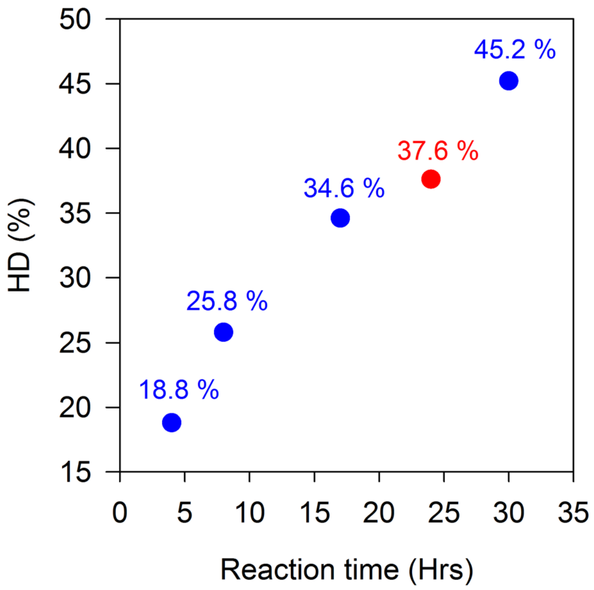

Figure S3 PEEK-OH hydroxylation degree (HD) resulting from the reaction of PEEK with $NaBH_4$ at various reaction times. Shown in red is the value for HD acquired in this work, whilst values shown in blue are those reported by Díez-Pascual and colleagues [1].

Equations

$$C = \frac{A}{Eb} \qquad \text{(Equation S1)}$$

$$M = 2.80\, mL\ x\ \left(C\ x\ \frac{1\,L}{1000\,mL}\right) \qquad \text{(Equation S2)}$$

$$C_{sample} = \left(\frac{M}{0.25\,mL}\right)\ x\ \frac{1000\,mL}{1\,L} \qquad \text{(Equation S3)}$$

Where: $C =$ -SH concentration as measured (mol L^{-1}), $E =$ TNB molar absorption coefficient, 14150 (M^{-1} cm^{-1}), b =path length of cuvette (1 cm), $M =$ -SH concentration as measured (mol), and C_{sample} = -SH concentration of the sampled volume 0.25 mL containing HA-SH (mol L^{-1}).

The concentration of –SH groups was converted from units of mol L^{-1} to mol g^{-1} by calculating the equivalent mass in grams of HA-SH sample in 1 L and dividing C_{sample} by this number (e.g. 20 g HA-SH in 1 L so 20).

$$M_{250^oC} = \left(\frac{M_s}{100}\right) x \,\% \, M_{250^oC} \quad \text{(Equation S4)}$$

$$M_{400^oC} = \left(\frac{M_s}{100}\right) x \,\% \, M_{400^oC} \quad \text{(Equation S5)}$$

$$M_{OH} = \% \, M_{400^oC} - M_{250^oC} \quad \text{(Equation S6)}$$

$$mol_{OH} = \frac{M_{OH}}{Mr_{OH}} \quad \text{(Equation S7)}$$

$$M_{PEEK-OH} = mol_{OH} \, x \, Mr_{PEEK-OH} \quad \text{(Equation S8)}$$

$$HD = \left(\frac{M_{PEEK-OH}}{M_{250^oC}}\right) x \, 100 \quad \text{(Equation S9)}$$

Where: M_s = TGA sample mass (g), M_{250^oC} = TGA sample mass at 250 ^{o}C (g), $\% \, M_{250^oC}$ = % TGA sample mass remaining at 250 ^{o}C (%), M_{400^oC} = TGA sample mass at 400 ^{o}C (g), $\% \, M_{400^oC}$ = % TGA sample mass remaining at 400 ^{o}C (%), M_{OH} = Mass of OH groups lost from PEEK-OH between 250 ^{o}C and 400 ^{o}C (g), mol_{OH} = Moles of OH groups lost from PEEK-OH between 250 ^{o}C - 400 ^{o}C (g), Mr_{OH} = Molar mass of OH group (g mol^{-1}), $M_{PEEK-OH}$ = Mass of PEEK-OH in sample (g), $Mr_{PEEK-OH}$ = Molar mass of PEEK-OH group (g mol^{-1}), and HD = PEEK-OH hydroxylation degree (%).

$$\sigma_f = \frac{3Fl}{2bd^2} \quad \text{(Equation S10)}$$

Where: σ_f= Flexural strength (MPa), F= Max force before yielding or fracture (N), l= Test specimen support separation length (mm), b= Test specimen width (mm), and d= Test specimen thickness (mm).

$$E_f = \frac{Fl^3}{4bd^3\delta} \qquad \text{(Equation S11)}$$

Where: E_f= Flexural modulus (MPa), F= Force (N), l= Test specimen support separation length (mm), b= Test specimen width (mm), d= Test specimen thickness (mm), and δ= Test specimen displacement (V_{DC}).

References

[1] Díez-Pascual AM, Martínez G, Gómez MA. Synthesis and Characterization of Poly(ether ether ketone) Derivatives Obtained by Carbonyl Reduction. Macromolecules. 2009;42(18):6885-92.
